# Calcite seed-assisted microbial induced carbonate precipitation (MICP)

**DOI:** 10.1371/journal.pone.0240763

**Published:** 2021-02-09

**Authors:** Jennifer Zehner, Anja Røyne, Pawel Sikorski

**Affiliations:** 1 Department of Physics, Norwegian University of Science and Technology (NTNU), Trondheim, Norway; 2 The Njord Centre, Department of Physics, University of Oslo (UiO), Oslo, Norway; VIT University, INDIA

## Abstract

Microbial-induced calcium carbonate precipitation (MICP) is a biological process inducing biomineralization of CaCO_3_. This can be used to form a solid, concrete-like material. To be able to use MICP successfully to produce solid materials, it is important to understand the formation process of the material in detail. It is well known that crystallization surfaces can influence the precipitation process. Therefore, we present in this contribution a systematic study investigating the influence of calcite seeds on the MICP process. We focus on the changes in the pH and changes of the optical density (OD) signal measured with absorption spectroscopy to analyze the precipitation process. Furthermore, optical microscopy was used to visualize the precipitation processes in the sample and connect them to changes in the pH and OD. We show, that there is a significant difference in the pH evolution between samples with and without calcite seeds present and that the shape of the pH evolution and the changes in OD can give detailed information about the mineral precipitation and transformations. In the presented experiments we show, that amorphous calcium carbonate (ACC) can also precipitate in the presence of initial calcite seeds and this can have implications for consolidated MICP materials.

## Introduction

Biocementation is a biological process, which improves the engineering properties of a granular medium by biomineralization. Biomineralized CaCO_3_ fills pores and bind the granular medium together, which results in an increase of strength and stiffness and reduce the permeability of the granular medium [1]. One of the most commonly used process to achieve biocementation is microbial induced calcite precipitation (MICP). MICP has been successfully used for soil stabilization [1, 2]. Using MICP for soil stabilization reduces the environmental impact of ground improvement, since conventional ground improvement techniques often have a high impact on the environment [3]. Moreover, MICP has the potential to replace conventional concrete as a building material in some applications. Possible applications include for example biodeposition and self-healing concrete [4]. Comparing concrete materials with same strength, MICP based concrete-like materials have the potential to lower the CO_2_ emissions of construction industry [5]. The production process of conventional cement, which is used as a binding material in convention concrete has a high CO_2_ emission and causes up to 5% of human-caused CO_2_ emissions [6]. MICP is commonly based on the urea hydrolysis reaction catalyzed by the urease enzyme [7, 8]:

$$\begin{array}{c} \text{CO}{\left({\text{NH}}_{2}\right)}_{2}+3{\text{H}}_{2}\text{O}\to 2{\text{NH}}_{4}{}^{+}+\text{HCO}3^{-}+{\text{OH}}^{-} \end{array}$$ (1)

The urea hydrolysis, catalysed by the urease enzyme, results in the production of ammonium and bicarbonate ions. Thereby, the production of the ammonium ions results in an increased pH of the solution. In the presence of sufficient concentration of calcium ions, urea hydrolysis can result in CaCO_3_ precipitation:

$$\begin{array}{c} {\text{Ca}}^{2+}+{\text{HCO}}_{3}{}^{-}+{\text{OH}}^{-}\to {\text{CaCO}}_{3}↓+{\text{H}}_{2}\text{O} \end{array}$$ (2)

Supersaturation is the driving force for precipitation, which can take place once the saturation state in the system *S* > 1 [9]:

$$\begin{array}{c} S=\sqrt{\frac{\left(\left(a_{Ca^{2+}}\right)\left(a_{CO_{3}^{2-}}\right)\right)}{K_{sp}}} \end{array}$$ (3)

where $a_{Ca^{2+}}\cdot a_{CO_{3}^{2-}}$ is the ion activity product of calcium and carbonate ions in the solution, and K_*sp*_ is the solubility product of the nucleating polymorph of CaCO_3_. A sufficient level of supersaturation results in nucleation and is followed by subsequent crystal growth. In MICP processes, the supersaturation is controlled among other factors, by the pH value, the amount of dissolved inorganic carbon and the calcium concentration (see Eq 3) [8]. In MICP, several injections of crystallization solution are typically used to achieve sufficiently consolidated granular material [10–12]. The crystallization solution consists of urea and a calcium source. As a source for urease, a urease-producing bacterium is added to the solution to catalyze the urea hydrolysis reaction. During initial injections, CaCO_3_ crystals will nucleate within the granular medium. Therefore, not only the granular medium, the bacteria cells, and gas bubbles in the system, but also CaCO_3_ crystals need to be considered in the material forming process. The presence of such surfaces in the crystallization solution results in heterogeneous nucleation. In heterogeneous nucleation the energy barrier necessary for nucleation is lowered and the nucleation rate increases [13].

The suitability of different surfaces as nucleation sites in heterogeneous nucleation has been investigated previously. Lioliou *et al*. [14] compared the nucleation of CaCO_3_ in the presence of calcite and quartz seeds and found that nucleation can take place at lower supersaturation levels for calcite seeds compared with quartz seeds. It was also shown by Dawe *et al*. that gas bubbles in the crystallization system promote CaCO_3_ precipitation [15]. Furthermore, the influence of bacteria cell surfaces as nucleation sites for MICP has been investigated. While Mitchell *et al*. showed that bacteria surfaces are not good nucleation sites [16], it has been shown recently by Ghosh *et al*. that nanoscale calcium carbonate crystals can form on the surface of *Sporosarcina pasteurii* [17]. *S. pasteurii* is an extensively studied microorganism for MICP processes [7, 18, 19].

We have previously reported an experimental method using absorption spectroscopy to study the precipitation process and the real time pH evolution of MICP without calcite seeds in volumes sufficiently small that mixing and transport occurred only by diffusion [20]. In this crystallization process, where no initial calcite crystals were present, three stages could be identified. In the first stage, urea hydrolysis catalyzed by bacterial urease increased the pH and during this phase precipitation of metastable amorphous calcium carbonate (ACC) and vaterite was observed (Stage I). After a short stable pH phase, nucleation of calcite was observed (Stage II). Nucleation and growth of calcite crystals resulted in a pH decrease (Stage III), while the ACC/vaterite was observed to dissolve.

To better understand the formation of a MICP consolidated material, it is important to know how the presence of CaCO_3_ crystals will influence the crystallization. Therefore, we have investigated the influence of calcite seeds on the crystallization process in MICP. We compare the precipitation in samples with and without calcite seeds and investigate the precipitation kinetics by monitoring the pH evolution in small volumes with absorption spectroscopy. From the changes in the optical density of the reaction solution, we determine the onset of the precipitation and follow phase transformations. We show, that the pH evolution is significantly different between unseeded and seeded samples and can give information about the precipitation process. Furthermore, we demonstrate that precipitation of ACC and vaterite can also occur for samples with calcite seeds present and that this process depends on the bacteria concentration and consequently on the urease activity.

## Materials and methods

### Chemical preparation

All solutions were filtered with a polycarbonate (0.22 μm) syringe filter before use. The used chemicals were purchased at Sigma-Aldich (Norway), unless otherwise stated. For preparation of the crystallization solution as well as for the bacteria culture, the same protocol as in previously presented work has been used [20] and a short description of the used protocols is given below.

#### Bacteria culture

For the presented experiments the urease-producing bacterium *Sporosarcina pasteurii* (Strain DSM33 of *S. pasteurii*) purchased from “Deutsche Sammlung von Mikroorganismen and Zellkulturen” (DSMZ) was used. DSMZ medium 220 with pH of 7.3 supplemented with urea and consisting of 15 g l^−1^ peptone from casein, 5 g l^−1^ peptone from soy meal, 5 g l^−1^ NaCl and 20 g l^−1^ urea was used as growth medium. The culture was inoculated with 1% frozen glycerol stock. After inoculation the culture was incubated overnight (17 h) at 30°C with constant shaking (200 rpm). Afterward, the culture was incubated without shaking at 30°C until further use, 24 h after inoculation. The cultures were prepared for the experiment by centrifuging the culture at 4800 ×*g* for 8 min and the cells were subsequently washed twice with pre-warmed 0.01 M PBS (phosphate buffered saline) to remove the growth medium. The cells were re-suspended and diluted with 0.01 M PBS. Three different bacteria cultures were used for the presented experiments. As an indication of bacterial cell concentration, the optical density (OD_600nm_) at 600 nm was measured in a 96-well plate (volume: 150 μm) before adding the bacteria suspension to the crystallization solution. The optical densities for the bacterial suspensions used in the presented experiments, as well as the used sample names are shown in Table 1.

**Table 1:** Bacteria cell concentrations of the cultures added to the crystallization solution.

Dilution name	OD_600nm_	Bacteria culture	sample name	
pH monitoring			unseeded	seeded
D1-C1	1.40	culture1	D1-C1_us_	D1-C1_s_
D2-C1	1.30	culture1	D2-C1_us_	D2-C1_s_
D3-C1	1.11	culture1	D3-C1_us_	D3-C1_s_
D4-C1	0.64	culture1	D4-C1_us_	D4-C1_s_
Optical microscope			unseeded	seeded
D2-C2	0.88	culture2	—	D2-C2_s_
D1-C3	0.89	culture3	D1-C3_us_	—
Confocal laser scanning microscope			unseeded	seeded
D1-C2	0.96	culture2	—	D1-C2_s_

Note: The table shows the name and the OD_600nm_ of the bacteria dilutions that were added to the crystallization solution. The dilution name indicates the dilution and bacteria culture that were used for the sample (for example: D1-C1 stands for Dilution 1- Culture 1). The ratio of bacteria dilution to sample volume was 1 to 10. Additionally, the table gives an overview of the sample names, which were used for unseeded and seeded sample.

The dilution of the original cultures with PBS is shown in S1 Table. The optical density values of bacteria cultures, given in Table 1, refer to the OD_600nm_ of the bacteria culture before adding the bacteria cells to the crystallization solution.

#### Crystallization solution

The crystallization solution for the presented experiments consisted of two components: urea, and dissolved chalk solution (DCS). The initial concentration of urea, before the start of the hydrolysis reaction, was 0.1 M. DCS was used as an environmentally friendly calcium source for MICP experiments. For the preparation of the DCS, 5 g of crushed limestone (Franzefoss Miljøkalk AS (Norway)) was dissolved in 50 ml of 300 mM lactic acid. After 24 h reaction time, the undissolved parts of the crushed limestone were filtered out with a 0.22 μm syringe filter. Urease producing bacteria (*Sporosarcina pasteurii*) cells were added to the crystallization solution to catalyze the hydrolysis reaction. For that, 20 μl bacteria dilution was added to 180 μl crystallization solution. The optical density of the bacteria suspensions is given in Table 1.

### Calcite seeds

Calcite seeds for optical microscopy and global pH monitoring experiments were prepared by mixing 0.2 M CaCl_2_ solution with 0.2 M Na_2_CO_3_ solution in an airtight reactor. The crystallization reaction was performed at 10°C and the solution was stirred with a mechanical stirrer for 48 h, following an established method [21]. After the reaction was completed, the calcite seeds were washed with water and ethanol before further use. The finished calcite seeds have been characterized with scanning electron microscopy. The calcite seeds had a rhombohedral shape with a width of about 8 μm (S1 Fig). The polymorphism of the seeds was confirmed with Raman microspectroscopy.

Additionally, for confocal laser scanning microscopy experiments, a fluorescent dye was incorporated in the calcite seeds during the crystal growth, following a procedure reported by Green *et al*. [22]. The fluorescent calcite seeds were directly grown on glass microscope cover-slides, as follows: The slides were placed in a beaker with CaCl_2_ solution and 0.1 nM solution of the fluorescent dye HPTS (8-Hydroxypyrene-1,3,6-trisulfonic acid). To initiate the crystallization, Na_2_CO_3_ solution was added and mixed thoroughly. The concentration of Ca^2+^ and CO_3_^2−^ was 5 nM. The reaction was left to proceed for 3 days. Afterwards, the slides were washed with DI water and ethanol before use. The shape of the crystals with fluorescent dye incorporated are shown in S2 Fig.

### Absorption spectroscopy

Real time pH evolution measurements were performed with a method reported earlier, to measure the average pH in small volumes (200 μL) [20]. Crystallization solution and the pH indicator Phenol Red (0.4 μM) were pre-mixed in a 96-well plate and bacteria suspensions were added to start the reaction. To minimize the gas exchange with the environment, the well plate was covered with a transparent tape before the measurement was started. The change in the optical density (OD) at 558 nm and 750 nm was measured with a spectrophotometer (SpectraMax^®^ i3 Platform). No signal from the pH sensitive dye was detected above 600 nm, meaning that the change in OD at 750 nm (OD_750nm_) was only caused by scattering of precipitates, seeds and bacteria cells in the sample. Consequently, the OD_750nm_ could be used for background corrections for the OD at 558 nm. The background corrected OD at 558 nm corresponds to the absorbance of the sample and the absorbance was used to calculate the pH, using a standard-curve created by recording the background corrected OD for calibration buffers with known pH. The OD_750nm_ was also used to monitor changes in amount of CaCO_3_ in the sample, since the change in OD_750nm_ due to bacterial growth is expected to be small compared to the change due to precipitation of CaCO_3_. For each bacteria cell concentration three parallel measurements were performed.

### Microscopy

#### Optical microscopy

The crystallization process with and without calcite seeds was monitored with an optical microscope (Motic, AE31E). The crystallization reaction was performed in a 96-well plate (sample volume: 200 μL). The objectives used were 10x (0.25 NA) for crystallization without seeds, and 20x (0.3 NA) for crystallization with seeds. Higher magnification was used for the seeded samples due to the small size of the calcite seeds. Images were recorded with a Moticam 5.0.

#### Confocal laser scanning microscopy

A confocal laser scanning microscope (CLSM, Leica TCS SP5) using an Argon laser (488 nm) and detection range 500 nm to 550 nm was used to investigate the crystal growth in the presence of fluorescent calcite seeds described above and to distinguish the initial calcite crystals and the microbial-induced CaCO_3_ precipitation.

#### Scanning electron microscopy

Scanning electron microscopy (SEM, Hitachi S-3400N) was used for characterizing the precipitated crystals. For SEM characterization the crystals were first dried on filter paper and the dry crystals were attached to carbon tape on the SEM stub. The crystals were sputter coated with a 10 nm layer of Pt/Pd (80/20) using Cressington 208 HR sputter coater. The acceleration voltage for the SEM imaging was 10 kV.

## Results

### Unseeded experiments

**Fig 1:**
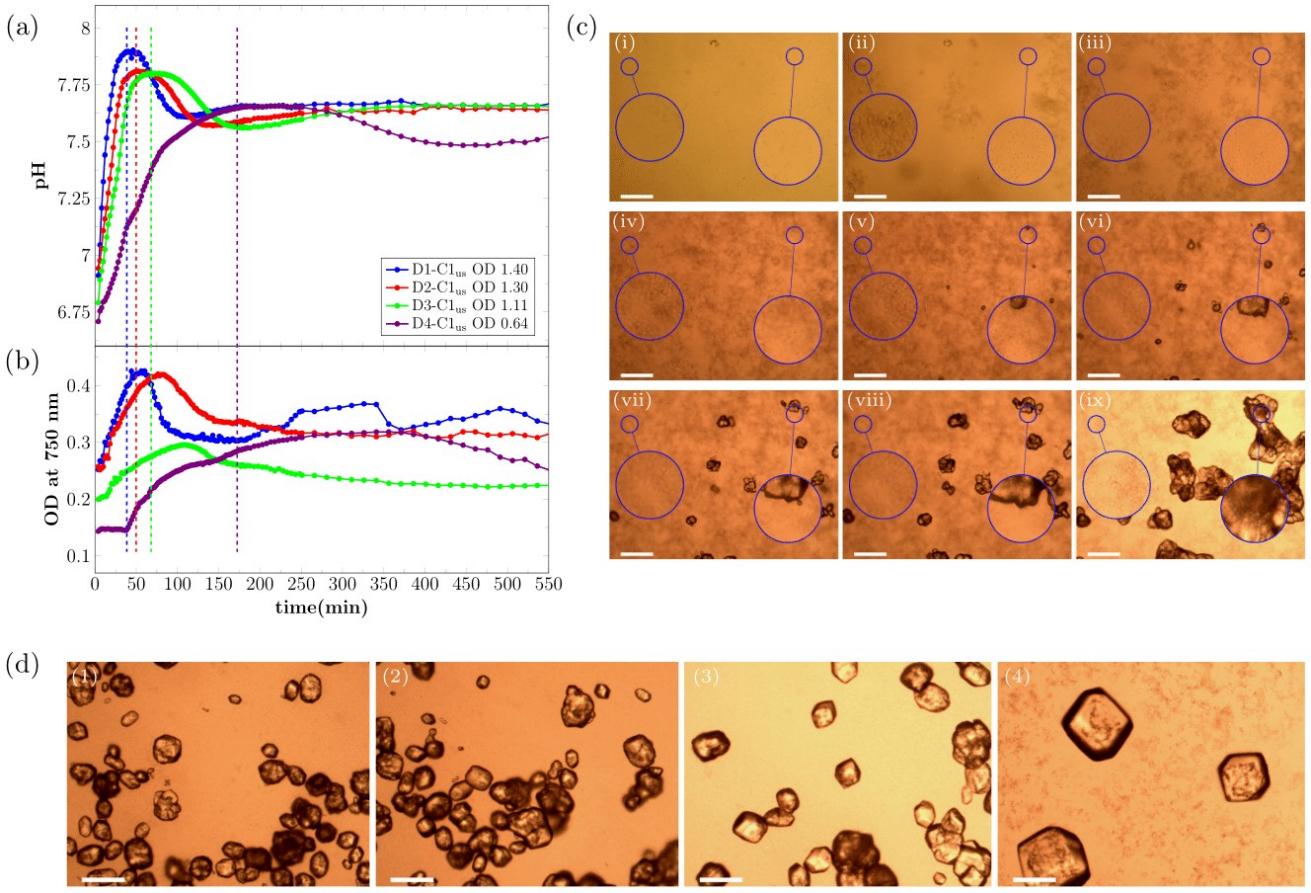
Real time evolution of (a) pH and (b) OD_750nm_ for MICP experiments without the presence of calcite seeds for the first 550 min of the reaction. Note that the first shown data point of the measurement is taken 4 min after the start of the reaction (= 0 min). The signal of the OD_750nm_ correlates with the amount of CaCO_3_ in the sample. Dashed vertical lines mark the time-point of maximum pH. The OD in the legend shows the OD_600nm_ of the bacteria dilution before adding to the crystallization solution. The error bars of the three parallel measurements in the real time pH evolution are in order of magnitude of the size of the shown data point symbols. (c) Optical microscope time-series of precipitation process for bacteria dilution D1-C3 (OD_600nm_ = 0.89) without calcite seeds present for different time-points of the reaction: (i) 0 min, (ii) 14 min, (iii) 23 min, (iv) 47 min, (v) 54 min, (vi) 88 min, (vii) 148 min, (viii) 240 min, and (ix) 21 h. The magnified area on the left shows an area where no calcite will nucleate during the experiment. The magnified area on the right shows an area where a calcite crystal will nucleate. (d) Micrographs of precipitated crystals after 21 h reaction time for (1) D1-C1_us_, (2) D2-C1_us_, (3) D3-C1_us_, and (4) D4-C1_us_ without the presence of calcite seeds. The scale-bar in (c) and (d) is 150 μm.

The pH evolution as well as the OD_750nm_ curves for CaCO_3_ precipitation, induced by hydrolysis of urea by *S. pasteurii*, in samples that did not contain calcite seeds are shown in Fig 1a and 1b. Experiments were performed with four different bacteria cell concentrations, as specified in Table 1.

As expected, the rate of the pH increase at the beginning of the reaction was correlated with the bacterial cell concentration and therefore with the urea hydrolysis rate (Fig 1a). The highest pH was reached for the samples that contained the highest bacterial cell concentration. After a short period of stable pH, the pH decreased and this trend was observed for all four tested bacterial cell concentrations. The pH remained stable longer for lower bacterial cell concentrations. Furthermore, it could be observed that the pH increased in the final stage of the reaction before stabilizing.

The observed changes in the pH of the crystallization solution were correlated with changes in OD_750nm_, which measures how much light is scattered by the sample. For the two highest bacteria cell concentrations, the OD_750nm_ increased fast from the beginning of the reaction and decreased after the maximum pH was reached in the sample. For the two lowest bacteria cell concentrations, the OD_750nm_ was nearly stable at the beginning of the reaction, before increasing after 14 min and 38 min for D3-C1_us_ and D4-C1_us_, respectively.

The increase in the OD_750nm_ during the first phase of the reaction, can be interpreted as precipitation of amorphous calcium carbonate (ACC), which quickly transformed into vaterite [20]. This was followed by the nucleation of calcite, accompanied by the dissolution of ACC/vaterite. The dissolution of the metastable phase resulted in a decrease of the OD_750nm_ for all four bacteria cell concentrations. This is a result of fewer, larger crystals scattering less light than many, small particles. The pH of the sample started to decrease once calcite nucleated (see Discussion and Fig 5 for more detailed description of the various processes taking place during precipitation). The second period of pH increase started at the same time as the OD_750nm_ signal started to stabilize. This second period of pH increase was most likely caused by a slowing down of the calcite precipitation rate due to a low amount of calcium remaining in solution.

This interpretation of the changes in pH and OD_750nm_ is supported by optical microscopy experiments. Fig 1c shows selected time-points for the precipitation reaction for the sample D1-C3_us_ (see Table 1). The precipitation of small particles was observed in the initial stage of the reaction (Fig 1c and 1ci–1civ). These precipitates have been characterized previously [20] and were identified as ACC, which quickly transforms to vaterite. After the nucleation of calcite crystals (Fig 1cv), the calcite crystals grew and the ACC/vaterite phase dissolved. The dissolution was fastest near existing calcite crystals (Fig 1c and 1cv–1cviii, magnified area on the right).

Fig 1d shows crystals precipitated in the experiments described above (Fig 1a and 1b), imaged after the reaction was completed (21 h). It was observed, that the bacteria cell concentration had a significant effect on the morphology of the precipitated crystals. For the highest bacteria cell concentration (D1-C1), the smallest crystals, with a mixture of rhombohedral and spherical morphology were observed (Fig 1d, 1). For samples with lower bacteria cell concentration, the precipitated crystals were larger and had a more regular, rhombohedral shape (Fig 1d, 2-4)).

### Seeded experiments

**Fig 2:**
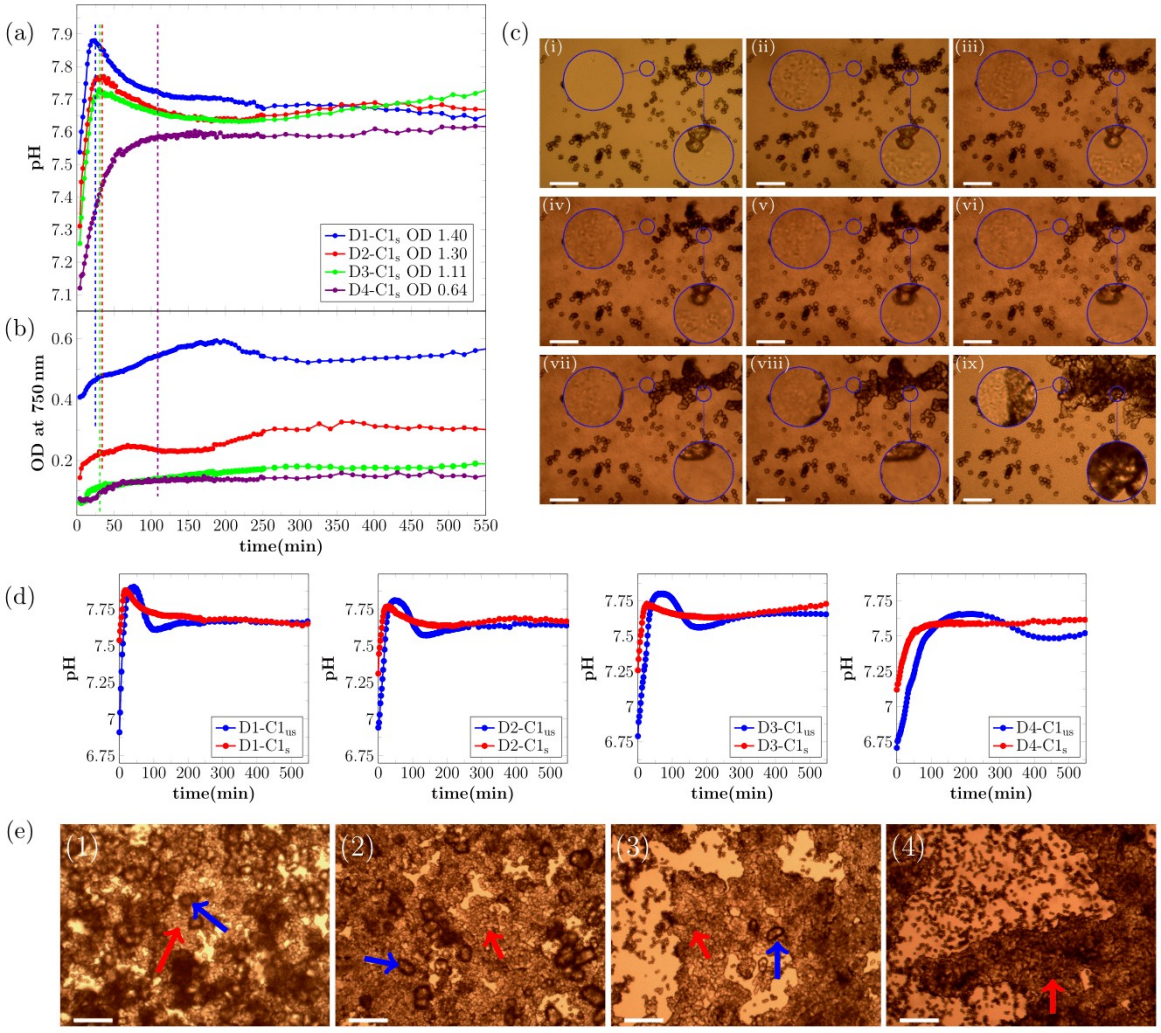
Real-time evolution of (a) pH and (b) OD_750nm_ for MICP experiments with the presence of calcite seeds. Note that the first shown data point of the measurement is taken 4 min after the start of the reaction. The OD_750nm_ was measured before start of the reaction to get the contribution of the initial seeds in the crystallization solution, and this starting value was subtracted from the OD_750nm_. Therefore, the shown signal is correlated to the amount of precipitated CaCO_3_. The OD in the legend shows the OD_600nm_ of the bacteria dilution before adding to the crystallization solution. (c) Optical microscope time-series of the precipitation process for bacteria dilution with OD_600nm_ = 0.88 with calcite seeds present for different time-points of the reaction: (i) 0 min, (ii) 20 min, (iii) 28 min, (iv) 50 min, (v) 80 min, (vi) 110 min, (vii) 170 min, (viii) 230 min, and (ix) 21 h. The magnified area on the right shows calcite seeds that will increase in size during the experiment. The magnified area on the left shows an area with a small distance to calcite seeds that will increase in size during the experiment. (d) Comparison of real time pH evolution of MICP experiments with and without calcite seeds. (e) Micrographs of precipitated crystals after 21 h reaction time for (1) D1-C1_s_, (2) D2-C1_s_, (3) D3-C1_s_, and (4) D4-C1_s_. The red arrows indicate calcite seeds that grew during the experiment and the blue arrows indicate additional nucleated calcite crystals. The scale-bar is 50 μm in (c) and 150 μm in (e).

The presence of calcite seeds had two effects on the MICP experiments (see Fig 2a and 2b). First, the seeds affected the pH of the crystallization solution, before the onset of the urea hydrolysis reaction, due to partial dissolution of the calcite seeds in the initially under-saturated solution. The starting pH was around 5.3 for unseeded and 6.1 for seeded samples (see S2 Table). Secondly, seeds affected the precipitation process, as the calcite seeds acted as nucleation sites for heterogeneous nucleation.

As for the unseeded samples, the pH increase at the beginning of the reaction was correlated to the bacterial cell concentration (Fig 2a). The period of rapid pH increase was followed by a pH decrease for the three highest bacteria cell concentrations. For the lowest bacteria cell concentration, no pH decrease was observed and the pH stabilized after the initial pH increase. The three parallel measurements for unseeded and seeded samples showed a derivation of not more than 0.05 pH units for each bacterial cell concentration.

As seen in Fig 2d, the shape of the pH curves is significantly different for unseeded and seeded samples. The initial pH increase had approximately the same slope for both unseeded and seeded samples at the start of the reaction. The offset along the time axis between unseeded and seeded pH curves was caused by the higher starting pH of the seeded samples (see S2 Table). The maximum pH was lower for the seeded samples. This was probably caused by an onset of nucleation and crystal growth at lower supersaturation levels due to the existing calcite surfaces. For unseeded samples, a stable pH phase at the maximum pH was observed, which was longer for lower bacterial cell concentrations. For the three highest bacteria cell concentrations, the pH decrease was faster for unseeded than for seeded samples. For the lowest bacterial cell concentration, no pH decrease was observed in the presence of calcite seeds (Fig 2d).

The OD_750nm_ of the seeded samples was measured before adding the bacteria cells and this value was subtracted from the subsequent real time OD_750nm_ measurement during the precipitation process to ensure the OD_750nm_ signal shown in Fig 2b does not include the contribution of the calcite seeds. Changes in the sample OD_750nm_ in Fig 2b were, therefore, mainly caused by the precipitation of CaCO_3_. For the two highest bacteria cell concentrations, the OD_750nm_ of the samples increased throughout the reaction. For the two lowest bacteria cell concentrations, the OD_750nm_ of the sample was constant at the beginning of the reaction and started to increase after 14 min and 28 min for D3-C1_s_ and D4-C1_s_, respectively. The increase in OD_750nm_ was observed for both samples only during the pH increase in the initial phase of the reaction.

The precipitation process for the sample D2-C2 (see Table 1) was investigated with optical microscopy (Fig 2c) to correlate changes in the pH and the OD_750nm_ to the precipitation process. Similar to the unseeded experiments, precipitation of small particles was observed in the initial phase of the reaction (Fig 2c, 2cii and 2ciii). The morphology of the precipitated particles was similar to what was observed in the unseeded experiments and, therefore, the initial precipitation could be identified as ACC/vaterite. The precipitation of ACC/vaterite was also indicated by a change in the OD_750nm_ of the samples during the initial reaction stage. The initial precipitation of ACC/vaterite was followed by growth of the crystal seeds present in the sample (Fig 2c and 2civ–2cix) and the ACC/vaterite dissolved at the later stage of the reaction (see magnified areas in Fig 2c and 2cv–2cvii), with faster dissolution in close proximity of the growing crystals (magnified area on the right). The dissolved ACC/vaterite reprecipitated on the surface of the seeds, but no pronounced decrease in the OD_750nm_ was observed, most likely due to the fact that less calcium was bound in metastable precipitates in seeded samples.

For the two lowest bacteria cell concentrations, the onset of precipitation occurred earlier in the seeded than in the unseeded experiments. This is likely a consequence of the higher starting pH of the crystallization solution, due to partly dissolved calcite seeds (S2 Table).

Optical micrographs of the precipitated crystals recorded after 21 h are shown in Fig 2e. For a low, only the growth on calcite seeds was observed (red arrow in Fig 2e, 4). However, for the three highest bacteria cell concentrations, additional nucleated calcite crystals were observed in the samples. The additional nucleated crystals are marked with a blue arrow and calcite seeds that increased in size during the reaction are marked with a red arrow (Fig 2e, 1-3). The additional precipitated calcite crystals in Fig 2e had a more irregular shape compared to samples without calcite seeds present (Fig 1).

**Fig 3:**
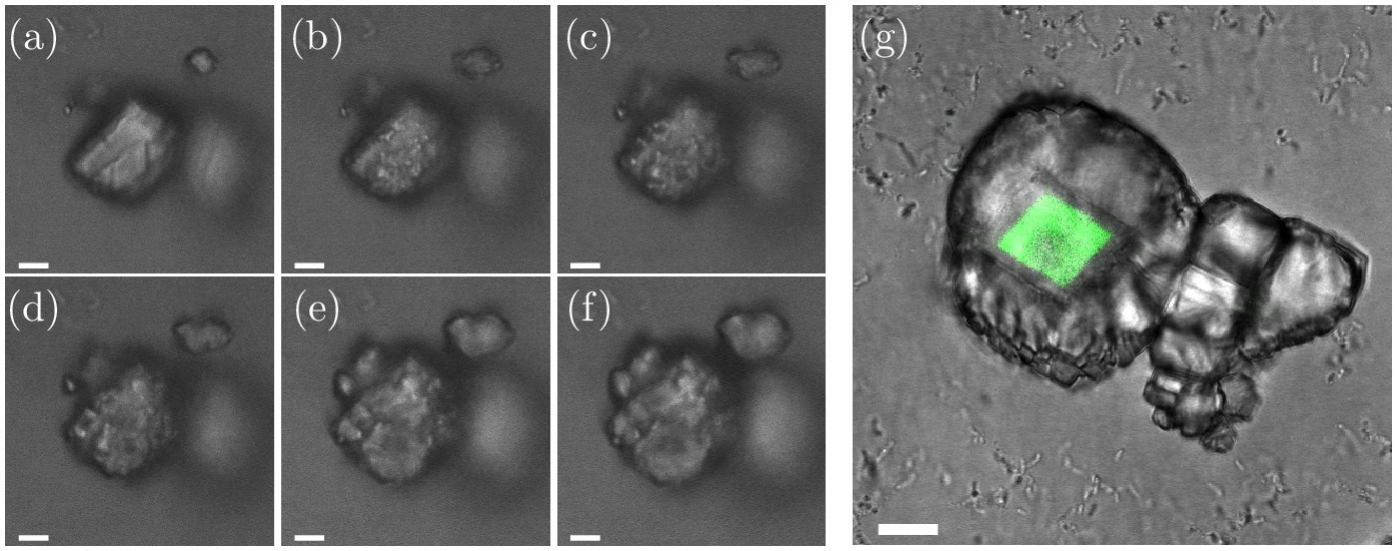
(a-f) Growth process of calcite seeds. Images are taken with a CLSM (confocal laser scanning microscope). Images are taken (a) 1 min before the growth process started, and (b) 1 min (c) 3 min (d) 6 min, (e) 11 min and (f) 14 min after the growth process started. (g) Finished crystal. The original fluorescent calcite seed (originated from an abiotic process) had a fluorescent dye incorporated and was visualized with CLSM. The scale-bar is 10 μm.

The growth process on the surface of the calcite seeds was investigated in more detail with confocal laser scanning microscopy (Fig 3a–3f). The growth process is shown for six time-points. Fig 3a shows the calcite seed 1 min before the crystal growth started. The crystal growth on the surface of the calcite seed started with small islands on the calcite crystal (Fig 3b) and the islands increased in size over the 13 min (Fig 3c–3e), until the growing crystals formed a new layer of calcite on the surface of the calcite seed (Fig 3f). The incorporation of a fluorescent dye into the initial calcite seed crystal (see Materials & methods) made it possible to distinguish between the initial crystal seeds (originated from an abiotic process) and the newly grown calcite crystals (originated from a biotic) on the seed surface (Fig 3g).

**Fig 4:**
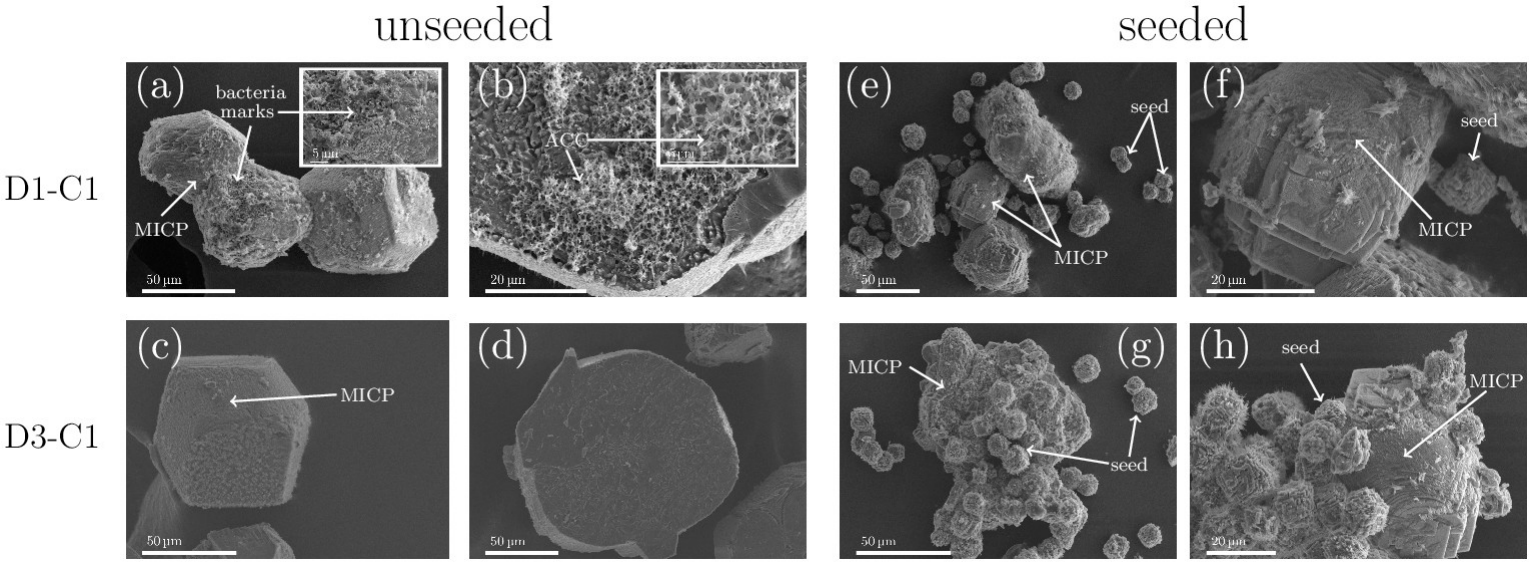
SEM characterization of precipitated crystals for unseeded and seeded samples for high bacterial concentration (D1-C1) and lower bacterial cell concentration (D3-C1). CaCO_3_ crystals that originated from MICP processes are marked with MICP in the SEM micrographs. (a) MICP induced calcite crystals for D1-C1 in unseeded experiments. Imprints of bacteria can be identified on the surface. (b) Bottom of the crystal for D1-C1 (unseeded), where the amorphous phase is enclosed in the calcite crystal. (c) MICP induced calcite crystals for D3-C1 in unseeded experiments and (d) the bottom of the crystals for D3-C1 in unseeded experiments, where no amorphous phase was detected. (e,f) Seeded samples for D1-C1. Initial calcite seeds (that did not grow during the experiment) and the MICP induced CaCO_3_ can be seen. (g,h) Initial calcite seeds (that did not grew during the experiment) and the precipitated CaCO_3_ can be seen. The initial calcite seeds can be identified within the precipitated CaCO_3_. Before SEM characterization, the crystals were transferred from the well plate to filter paper for drying. The crystals were not washed before SEM characterization. Needle-like crystals on the calcite surfaces are most likely calcium lactate, as this phase is expected in the precipitated samples.

Dried crystals for unseeded and seeded samples were characterized with SEM (Fig 4).

For the unseeded samples, it can be seen that the precipitated calcite crystals are bigger for the lower bacterial cell concentration (Fig 4a and 4c). Furthermore, for D1-C1, the imprints of bacterial cells as well as bacterial cell can be identified on the crystal surface (small insert in Fig 4a). Due to the lower bacterial cell concentration less bacterial imprints and bacterial cells could be detected on the surface of the calcite crystal of D3-C1 resulting in a smoother crystal surface (Fig 4c). Small, needle shape crystals on the surface of the crystal are most likely calcium lactate crystals, since the crystals were not washed before the drying and characterization process, and this phase is expected in the precipitated samples. Furthermore, the bottom side of the crystals was analyzed for the unseeded samples (Fig 4b and 4d). The bottom-side of the crystals is the part of the crystals that was growing on the well surface. Here we could detect, that for the highest bacterial cell concentration (Fig 4b), the amorphous phases was encapsulated by the growing crystal. This was most likely caused by the fact that a fast growth process isolated some amount of the initially precipitated amorphous phase from the crystallization solution. Since the transformation process of the amorphous phases to calcite is a dissolution based transformation, the transformation of the amorphous phases was suppressed. No amorphous phases were detected on the bottom of the crystal for the lower bacterial cell concentration (Fig 4d). SEM images of ACC have been reported previously [23, 24] and are in good agreement with the morphology of the amorphous phase, shown in Fig 4b. For the seeded samples the initial calcite seeds as well as seeds which grew during the experiments could be observed in the SEM micrographs (Fig 4e–4h). For D1-C1 seed crystals which grew during the experiment could not be identified due to precipitation at high supersaturation (Fig 4e and 4f). For the lower bacterial cell concentration, the initial calcite seeds (originated from an abiotic process) and the precipitated crystals (originated from MICP process) could be distinguished (Fig 4g and 4h). It can be seen, that the precipitated crystals grew on the surface of the calcite seeds and connecting them together (Fig 4g). Furthermore, initial calcite seeds have a different surface compared to the MICP induced calcite, as can be seen in Fig 4h.

## Discussion

In the presented experiments, we investigated the influence of calcite seeds on the CaCO_3_ precipitation in MICP processes.

**Fig 5:**
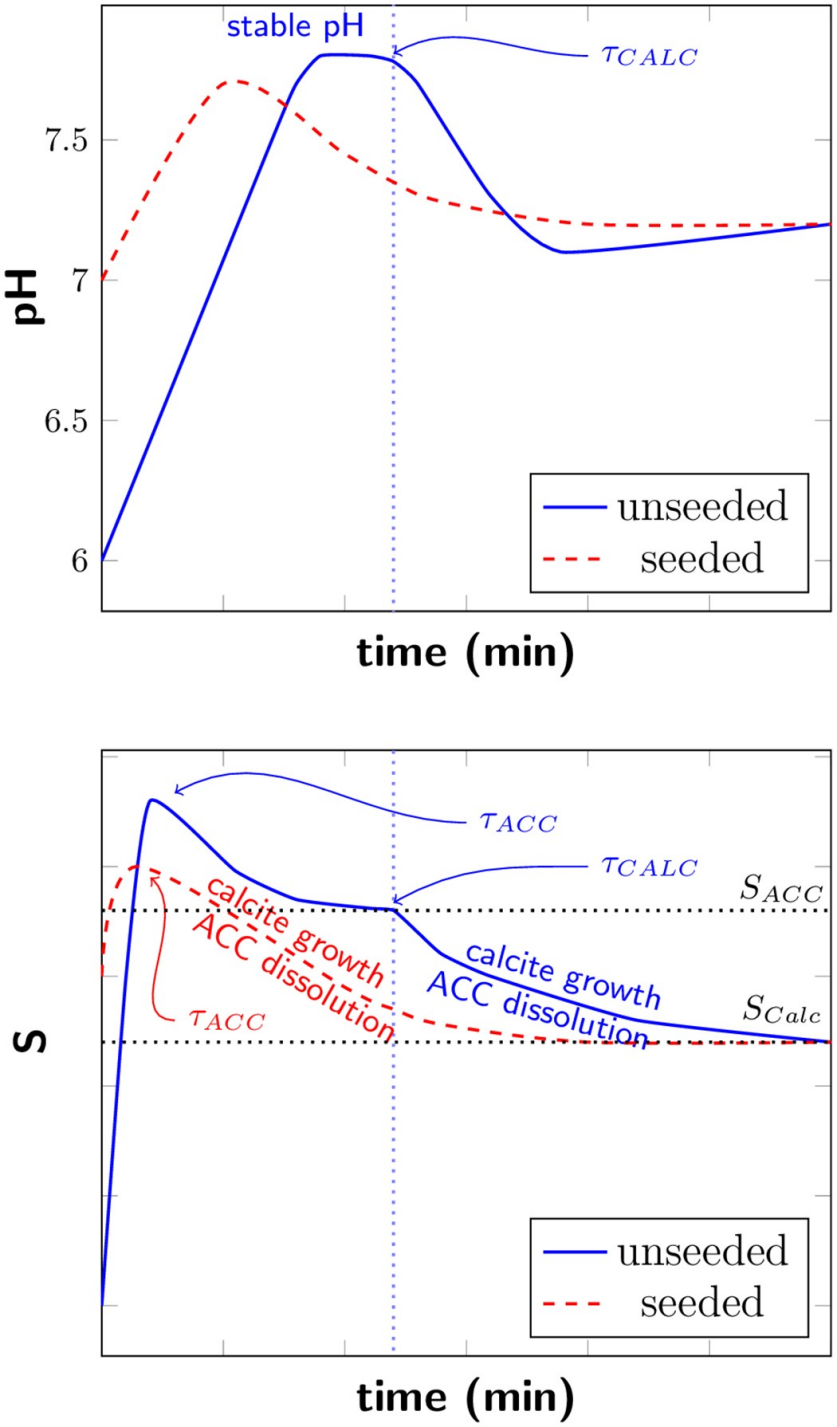
Schematic illustration of real time pH evolution (top) and saturation level (bottom) of unseeded and seeded samples with high bacteria cell concentration. *τ*_*ACC*_ and *τ*_*CALC*_ mark the nucleation of ACC and calcite. *S*_*ACC*_ and *S*_*CALC*_ represent *S* = 1 with respect to ACC and calcite, respectively.

We observe significant differences in the pH evolution for unseeded and seeded samples (Fig 2d). These differences can be connected to the saturation state in the sample and consequential to different processes during the precipitation. A schematic illustration of the connection between the saturation state and the pH evolution for unseeded and seeded samples with high bacterial cell concentration is shown in Fig 5.

In the starting phase of the experiment, a rapid pH increase was observed for both unseeded and seeded samples. With increasing pH, also the saturation in the sample is increasing. The pH increase was correlated to the urea hydrolysis rate. The bacteria cultures were cultivated following a standardized protocol and we have therefore not measured the urea hydrolysis rate directly. However, the bacteria cell concentration is directly connected to the urea hydrolysis rate [25] and was therefore used as a measure for the urea hydrolysis rate. The bacteria cells were washed before the experiments, therefore, there was no free enzyme present in the bacteria dilutions. However, the used bacteria strain (DSM33) has always urease enzyme with high activity present [26, 27], located most likely in the cell membrane. Therefore, urea hydrolysis and the pH increase started just after adding the bacteria to the sample well, despite the fact that the timescale of the experiment was not sufficiently long for the bacteria to produce urease as a response to the urea in the sample.

For unseeded samples, ACC nucleated (*τ*_*ACC*_) once the saturation *S* was sufficiently above *S*_*ACC*_ (*S*_*ACC*_ represent *S* = 1 with respect to ACC) and the precipitation of ACC reduced *S*. Precipitated ACC transformed rapidly to vaterite, according to Ostwald’s rule of stages (for simplification, this transformation is not shown in Fig 5). When the stable pH was reached, the urea hydrolysis rate by bacterial urease was equal to the precipitation rate. In this stable pH region, the saturation level *S* is reduced to *S*_*ACC*_ due to the growth of the ACC phase and stays constant until the nucleation of calcite occurs, as schematically illustrated in Fig 5. Once calcite nucleated, nucleation and crystal growth of calcite contributed to reduce *S* and resulted in a pH decrease. The solution became under-saturated with respect to ACC/vaterite and the metastable phases dissolved.

With decreasing bacteria cell concentration, and therefore decreasing urea hydrolysis rate, a lower maximum pH values and correspondingly lower *S* were reached in the starting phase of the reaction. For lower *S*, it takes longer until calcite nucleates and consequently a longer stable pH region was observed before the pH decreased (Fig 1a).

For the seeded samples, a short stable pH region was observed for the three highest bacterial cell concentrations and the shape of the pH curves was similar for those samples. For the lowest bacterial cell concentration, no pH decrease was observed and the pH stabilized after the initial pH increase (Fig 2a). The difference in the pH evolution for seeded samples could be connected to the precipitation of ACC and calcite. For the three highest bacteria cell concentrations, the pH increased until a short stable pH phase was reached, before the pH decreased. The pH decrease indicated that nucleation and growth of the calcite seeds was not sufficient to consume hydrolysis products. High pH and corresponding high supersaturation resulted in precipitation of ACC, also for seeded samples. This was also confirmed by optical microscopy (Fig 2c). The pH decrease was a consequence of nucleation of calcite crystals and crystal growth. The initial calcite seeds acted as nucleation sites, and therefore no significant pH plateau was observed before calcite nucleation. With decreasing pH and therefore decreasing *S*, ACC/vaterite dissolved (see Fig 5, red dashed line). For the lowest bacterial cell concentration, no pH decrease was observed. Due to a lower urea hydrolysis rate, caused by a lower bacteria cell concentration, a lower saturation level was reached. In this case, the supersaturation produced by urea hydrolysis was most likely reduced by growth of existing calcite seeds and no ACC precipitated for the seeded sample with the lowest bacterial cell concentration. ACC/vaterite precipitation was reported earlier for unseeded samples [20, 28]. However, we were able to show by comparing unseeded to seeded experiments that the metastable polymorph phases can also precipitate for samples where calcite seeds were present (Fig 2). This could also be confirmed by analyzing the shape of the pH evolution curves. The presence of calcite seeds lowered the energy barrier for nucleation, resulting that lower pH values were necessary for calcite nucleation. However, the presence of calcite seeds did not suppress the formation of metastable phases for higher bacteria cell concentrations.

Calcite nucleation and growth on the seeds started at lower pH values in seeded samples (Fig 2d) than the calcite nucleation in unseeded samples and therefore at lower supersaturation. The influence of calcite seeds on CaCO_3_ precipitation in non-MICP experiments was reported earlier and it was shown, that in the presence of calcite seeds, CaCO_3_ nucleation can take place at lower supersaturation levels [14, 29]. In our experiments, we could confirm this also for MICP. Furthermore, the calcite seeds had a significant influence on the precipitation process. With SEM characterization (Fig 4e–4h), we could show that the precipitated CaCO_3_ connected the initial seeds together.

Furthermore, it was observed for unseeded samples that the bacterial cell concentration had a strong influence on the size and shape of the precipitated calcite crystals. It has been reported previously that a high supersaturation condition for nucleation leads to smaller CaCO_3_ crystal sizes [30]. Cheng *et al*. and Cuthbert *et al*. reported that the initial ureolysis rate in MICP experiments influenced the crystal size [31, 32]. Wang *et al*. showed recently that higher bacteria densities in MICP experiments resulted in a high nucleation rate, due to the precipitation of unstable phases of CaCO_3_ [25]. This was also observed in our experiments (Figs 1d, 4a and 4c). A higher bacteria cell concentration in the beginning of the experiment resulted in a larger amount of ACC/vaterite precipitation, which could act as nucleation sites for calcite crystals. This resulted in the nucleation of a larger number of smaller crystals.

## Conclusion

We presented a systematic study investigating the influence of calcite seeds on the crystallization processes of CaCO_3_ in MICP. In our experiments we observed a significant difference in the pH evolution between unseeded and seeded experiments, caused by different precipitation processes of CaCO_3_. By microscope analysis and analyzing the shape of the real time pH evolution and the OD_750nm_ we could detect the precipitation of the metastable phases (ACC/vaterite), also for samples with calcite seeds present. Furthermore, we detected that lower pH values were necessary for calcite nucleation in seeded experiments. We showed that the precipitation of metastable precipitates in seeded samples is connected to the bacterial cell concentration and therefore to the urea hydrolysis activity.

The precipitation of metastable phases is important in biocementation processes, as it might affect the homogeneity of the consolidated material. ACC particles are small and have a lower density compared to calcite and could therefore be transported with the flow through the granular medium. Small pores in the granular medium can be clogged with ACC and therefore influence the homogeneity of calcite precipitation. The results obtained in this study show that, also in the presence of initial calcite crystals, ACC can precipitate for high bacterial cell concentrations and that no ACC precipitates in seeded samples with low bacteria cell concentrations. This needs to be considered in MICP protocols to avoid clogging of the granular medium and to achieve a knowledge-based improvement of MICP materials.

## Supporting information

S1 Fig SEM images of calcite seeds. (TIF)

S2 Fig Confocal laser scanning microscope images of fluorescent calcite seeds grown on glass cover-slides. (left) Brightfield images of calcite seeds and (right) combination of brightfield image and fluorescent signal of incorporated fluorescent dye.
(TIF)

S1 Table Dilution of original bacteria cultures. The original culture with growth medium was centrifuged, washed and re-suspended in 0.01 M PBS. The re-suspended bacteria cultures without growth medium were dilute to the final dilutions, which were used for the experiments.
(TIF)

S2 Table Starting pH value of the crystallization solution for samples with and without calcite seeds present before adding the bacteria culture to the crystallization solution. (TIF)
