# Supplementary figures and images for: Calcite seed-assisted microbial induced carbonate precipitation (MICP)
Source: PLoS One. 2021 Feb 9;16(2):e0240763. doi: 10.1371/journal.pone.0240763 (PMC7872276; doi:10.1371/journal.pone.0240763)

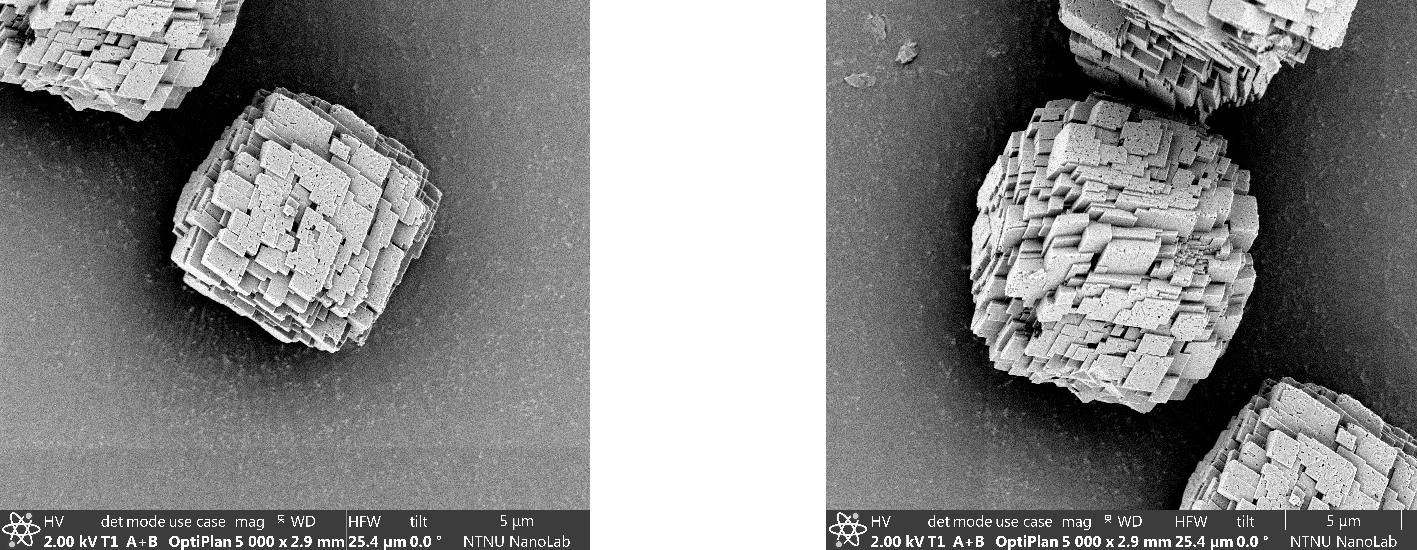

Supplement: S1 Fig — (TIF) [file pone.0240763.s001.tif]

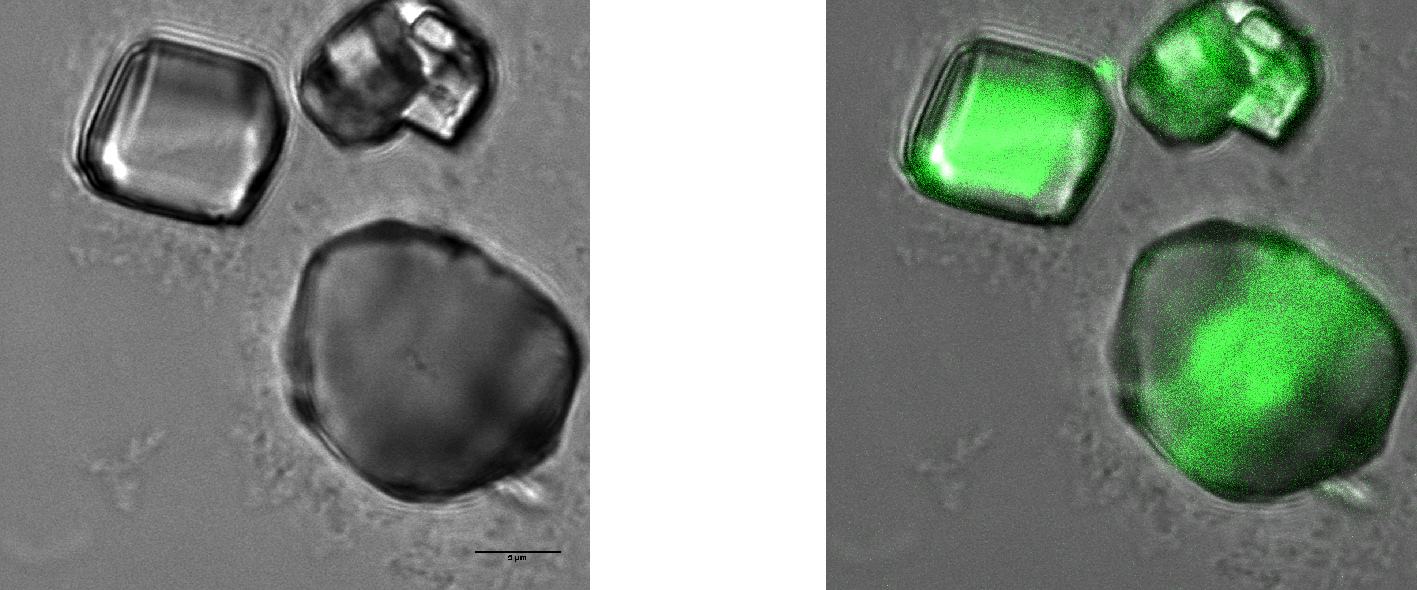

Supplement: S2 Fig — (left) Brightfield images of calcite seeds and (right) combination of brightfield image and fluorescent signal of incorporated fluorescent dye. (TIF) [file pone.0240763.s002.tif]

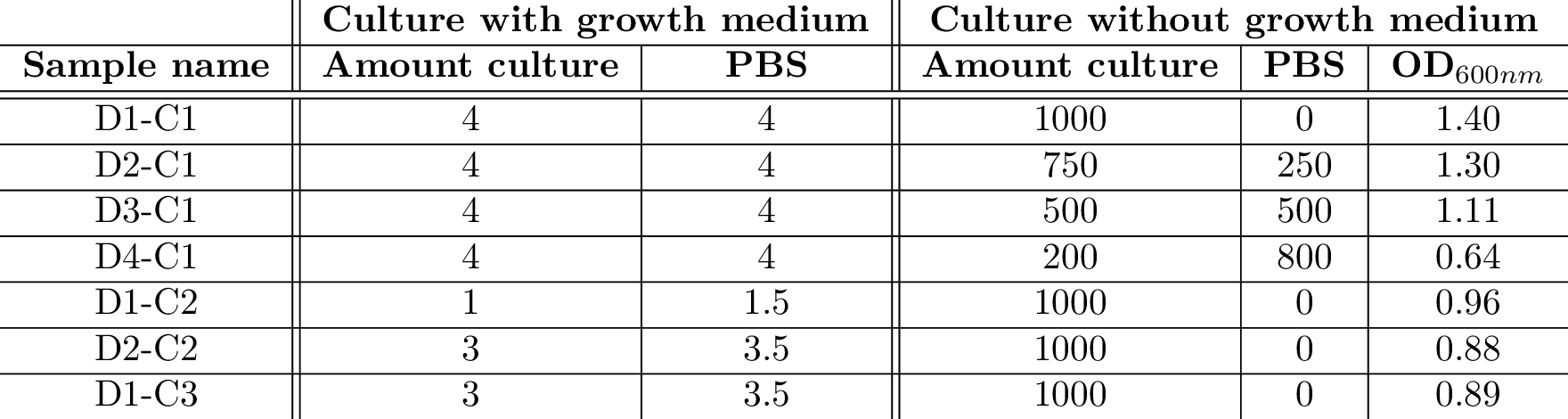

Supplement: S1 Table — The original culture with growth medium was centrifuged, washed and re-suspended in 0.01 M PBS. The re-suspended bacteria cultures without growth medium were dilute to the final dilutions, which were used for the experiments. (TIF) [file pone.0240763.s003.tif]

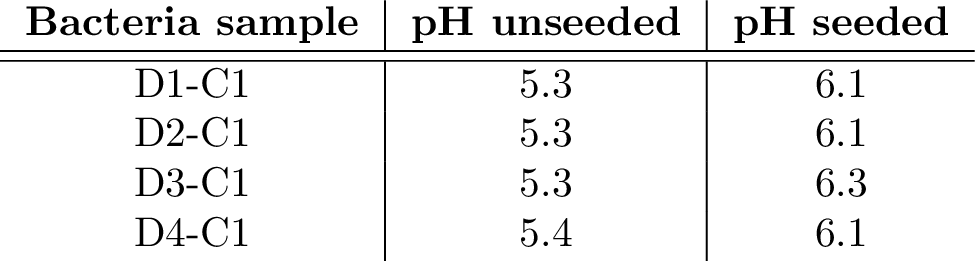

Supplement: S2 Table — (TIF) [file pone.0240763.s004.tif]
